# Possession in motion: a five-season analysis of running in-possession and out-of-possession with match outcomes in the English Premier League

**DOI:** 10.5114/biolsport.2026.159531

**Published:** 2026-02-06

**Authors:** Tom Allen, Matt Taberner, Mikhail Zhilkin, Damian Harper, Jill Alexander

**Affiliations:** 1 Arsenal Performance and Research Team, Arsenal Football Club, London, UK; 2 Football Performance Hub, Institute of Coaching and Performance (ICaP), School of Health, Social Work and Sport, University of Central Lancashire, Preston, UK; 3 Research Institute of Sport and Exercise Sciences, Liverpool John Moore’s University, Liverpool, UK

**Keywords:** Elite football, Success, Technical, Running load, High-intensity running

## Abstract

This study examined relationships between running load, possession metrics, and team success (points per game (PPG)), across five English Premier League (EPL) seasons (2019/20–2023/24). Comparison of running and possession demands when facing top six (T6) versus lower-ranked (7–20) opponents was also explored. Analysis of 1675 matches, using Second Spectrum^®^ optical tracking system, to measure running load variables (total distance (TD), high-intensity distance (HID), high-speed running (HSR), sprint distance (SprD), expressed as totals, in-possession per minute (TIP), out-of-possession per minute (TOOP), and TOOP/TIP ratios. Pearson’s correlations assessed relationships between running load, possession, ball-in-play (BiP), and PPG. Differences between T6 and lower-ranked teams were assessed using Wilcoxon rank-sum tests. PPG correlated very largely with possession (r = .74, p < 0.001), and largely with TOOP/TIP ratios and BiP (r = .50–.65, p < 0.001). Moderate positive correlations with PPG were observed with TOOP metrics (r = .34–.45, p < 0.001), while SprD showed a small positive relationship (r = .25, p=0.01). TIP metrics correlated negatively with PPG (r = –.22 to –.57, p = 0.001–0.023). Against T6 teams, clubs ran more in total and TIP (ES = 0.11–0.41, p < 0.02), but less TOOP and lower TOOP/TIP ratios (ES = –0.11 to –0.54, p < 0.006), alongside reduced possession (ES = –0.96 to –1.04, p < 0.001) and PPG (ES = –0.61 to –0.66, p < 0.001). EPL success is very largely associated with possession and greater out-of-possession running, while in-possession running relates negatively. Only SprD correlated with PPG, suggesting total running load is less influential than possession and defensive running efficiency.

## INTRODUCTION

Understanding performance in elite football requires integrating technical, tactical, and physical components, as match outcomes emerge from the interaction of these factors [1–3]. Modern match analysis therefore increasingly emphasises the contextual interpretation of running performance; how, when, and why players produce physical actions within specific tactical situations [3] and how it may impact team success [4].

When a team has possession of the ball in football, it must use possession efficiently to create scoring opportunities. Research consistently indicates that possession distinguishes top-performing teams from lower-ranked ones in competitive football [5–7]. Across major European leagues, higher-ranked teams consistently exhibit greater possession [8], leading to more attempts on goal [9], increasing the chances of successful match outcome [10]. Possession also reflects broader tactical intentions: stronger teams often sustain longer attacking phases, whereas weaker teams frequently concede possession to protect space nearer their own goal by adopting deeper defensive blocks [11]. However, possession alone offers only partial insight into performance without considering the physical and tactical actions that underpin it.

Running load outputs in the English Premier League (EPL) have been reported to be increasing [12–13]. Yet physical output alone does not fully explain team success, and small-to-moderate associations between running load and performance [4, 14] suggest that the value of physical actions is heavily shaped by tactical context [4]. Contextual analyses have further shown that high-speed running (HSR) out-of-possession is sensitive to opponent ranking and match location, decreasing as ranking difference increases and increasing during away matches [3]. Therefore, a holistic approach that integrates technical, tactical and physical elements is essential [15]. Several studies have therefore examined running with and without the ball. Higher possession teams tend to cover greater high-intensity distance (HID) when in-possession [16] and running with the ball has been linked to higher league ranking in multiple European leagues [17–19]. Conversely, successful teams in UEFA Champions League matches have been shown to exhibit higher running intensities out-of-possession than in-possession [20], suggesting that defensive and transitional running behaviours may be more indicative of team effectiveness.

A key limitation of existing research is that most studies report total distances, which are strongly influenced by possession percentage, match duration, and ball-in-play (BiP) time [21]. Consequently, totals may obscure the tactical nature of running demands. Expressing running load relative to time spent in-possession (TIP) and out-of-possession (TOOP), using metres per minute (m · min^−1^), provides a more precise indication of physical intensity within distinct tactical phases. Recent findings highlight relative intensities provide a more valid representation of match demands than totals alone, due to the influence of possession phases and formation structures on players’ running opportunities [3, 22]. Despite growing interest in tactical–physical interactions, no study to date has examined possession-related running intensities in the English Premier League (EPL) using m · min^−1^ metrics, nor has any work evaluated the balance of TOOP and TIP intensities (TOOP/TIP ratios) as potential performance indicators. Metres per minute is required because teams spend different amounts of time in- and out-of-possession. Without normalising for possession time, high-possession teams appear to run more simply because they have the ball for longer. Using m · min^−1^ for TIP and TOOP provides a relative measure of intensity that reflects tactical behaviour within each phase and allows meaningful comparisons between teams with different possession styles. This helps identify tactical tendencies, such as speed of build-up play, counter pressing tendencies, or defensive compactness; all of which total distances cannot capture.

Furthermore, little is known about how these metrics vary according to opposition strength. Tactical behaviour is known to adjust based on opponent quality [23–24], yet it remains unclear whether the relationships between running load, possession, and performance differ when teams face higher-ranked versus lower-ranked opponents in the EPL.

Therefore, the primary aim of this study was to analyse the relationships between possession and team running load metrics (total distance (TD), HSR (speeds between 5.5 ms^−1^ – 7 ms^−1^), HID (> 5.5 ms^−1^), and sprint distance (SprD; > 7 ms^−1^), measured in total and per-minute (TIP, TOOP), and team performance measured as points per game (PPG) across five EPL seasons. A secondary aim was to compare running, and possession demands when teams faced top six opponents versus lower-ranked teams, to determine whether tactical and physical behaviours differ in relation to opposition quality.

## MATERIALS AND METHODS

### Participants

Team running load and possession outputs across five consecutive EPL seasons (season 2019/20 to season 2023/24) were used for analysis (Table 1). Ethical approval for the use of match data was obtained from the EPL and host university (BAHSS2 0327).

**TABLE 1:** Number of games that finished 11 v 11, mean game duration, players included in that season’s analysis and mean age of players that season.

Season	Games that finished 11 vs 11	Mean Game Duration	Players Included in Analysis that Season	Mean Age of Players
2019/20	333	97.4 ± 1.8	515	27.0
2020/21	336	96.4 ± 2.1	525	27.0
2021/22	336	97.2 ± 2.3	538	27.2
2022/23	346	98.4 ± 2.8	555	27.0
2023/24	324	101.2 ± 2.8	571	26.7

### Procedures

Optical tracking data was collected from EPL stadiums using Second Spectrum^®^ (2S; Los Angeles, USA). The dwell time (minimum effort duration) was set at 0.5 s for HID running and 1 s to detect SprD efforts. All player data was anonymised prior to analysis. Games which finished with less than 22 players on the pitch (216 through sending offs or injury, 9 due to an inability to replace an injury) were removed from the data set. Once the inclusion criteria were met, the data set included 26 teams with 1196 players used from 1675 games over the five seasons.

Running activities investigated included TD (m), HSR (m; 5.5 ms^−1^ – 7 ms^−1^), HID (m; > 5.5 m · s^−1^), which includes HSR and SprD (> 7.0 m · s^−1^), and SprD (m). These running load measurements were chosen as 2S^®^, has been tested and certified under the FIFA Electronic Performance & Tracking Systems Quality Programme for positional and velocity accuracy in football-specific movements [25]. allowing comparison through the seasons. All running outputs were measured as totals (volume; total running completed in the game) and per minutes (intensity; total running / total match duration) when the team were in-possession (TIP; running variables in this time period / TIP time) and when the team were out-of-possession (TOOP; e.g. distance covered during TOOP / time out-of-possession = TD/min TOOP). Possession time was calculated directly from the tracking data, allowing running outputs to be expressed relative to the actual time each team spent in- and out-of-possession (i.e. 2000 m run in 20 mins TOOP = 100 m/min when TOOP). These measures were also observed as a ratio to determine whether a team had a higher intensity in or out-of-possession in certain physical metrics. All team-season values therefore represent the average or total running outputs aggregated across all matches that season, rather than match-by-match observations. Goalkeepers were included in the team-level analysis, consistent with recent work examining team running trends [12–13] and the increasing tactical involvement of goalkeepers in build-up and defensive organisation [26]. Technical variables measured were, possession percentage and ball in play (BiP) time, with PPG being used to determine team success.

### Statistical Analysis

Data was collected to observe the association between running load in and out-of-possession and PPG over the five seasons (Table 2). Analyses were conducted using team season averages, meaning each team contributed one value per variable per season. This approach limits repeated measures requirements and avoided match level nesting. Our aim was to describe broad, between team relationships rather than model within team changes over time, so Pearson correlations were utilised for this exploratory analysis. As such, the findings should be interpreted as cross-sectional associations rather than causal effects. Further comparisons were made for the top six teams (T6) over the five-season period. T6 was determined as the six teams with the highest points that season (by points gained that season in all games, see Table 3), providing a consistent indicator of EPL performance and reducing ambiguity introduced by cup results or alternative qualification pathways. Team performance was then used to compare the T6 and the other 14 teams to see if there was a difference in-possession and running load outputs. This was replicated for each season of analysis.

**TABLE 2:** Points per game (PPG), average possession percentage per EPL game, ball in play (BiP) minutes per game and running load outputs when in and out of possession for teams who had played in the EPL between 2019/20 and 2023/24. Data is ordered by points per game

Team	Number of seasons in Data Set	PPG	Possession %	BiP minutes / game (% of game)	TD/min TIP	TD/min TOOP	TD TOOP/TIP ratio	HID/min TIP	HID/min TOOP	HID TOOP/TIP ratio	SprD/min TIP	SprD/min TOOP	SprD TOOP/TIP ratio
Manchester City	5	2.37	64%	63%	123.0	150.9	1.23	8.77	18.70	2.14	2.09	4.15	1.99
Liverpool	5	2.15	59%	59%	129.2	149.0	1.15	11.80	18.30	1.55	3.18	3.83	1.20
Arsenal	5	1.91	56%	57%	134.0	147.3	1.10	11.10	16.70	1.51	2.73	3.71	1.36
Manchester United	5	1.76	53%	58%	130.6	142.9	1.09	12.70	16.10	1.27	3.51	3.46	0.99
Chelsea	5	1.65	58%	58%	129.8	144.6	1.11	11.50	16.60	1.45	3.00	3.65	1.22
Tottenham Hotspur	5	1.62	53%	58%	132.0	147.2	1.11	12.40	16.00	1.29	3.27	3.26	1.00
Leicester City	4	1.45	51%	57%	130.8	146.8	1.12	11.30	15.80	1.40	2.88	3.21	1.11
Newcastle United	5	1.43	45%	55%	135.8	144.5	1.06	13.40	15.70	1.17	3.69	3.39	0.92
Aston Villa	5	1.38	49%	54%	130.7	143.5	1.10	12.20	15.70	1.28	3.23	3.38	1.05
West Ham United	5	1.35	43%	58%	136.2	138.5	1.02	13.60	13.30	0.98	3.14	2.63	0.84
Wolverhampton Wanderers	5	1.3	49%	57%	130.7	138.2	1.06	12.20	13.60	1.12	3.36	2.83	0.84
Brighton and Hove Albion	5	1.28	55%	58%	135.5	146.1	1.08	11.40	16.20	1.42	2.68	3.44	1.28
Everton	5	1.22	45%	56%	137.2	144.5	1.05	13.50	15.20	1.13	3.48	3.10	0.89
Brentford	3	1.21	45%	54%	138.0	147.0	1.07	12.90	16.70	1.30	3.23	3.52	1.09
Crystal Palace	5	1.17	46%	56%	130.7	140.6	1.08	10.80	13.70	1.28	2.59	2.63	1.02
Fulham	3	1.12	50%	57%	133.2	142.4	1.07	12.70	15.10	1.19	3.40	3.14	0.92
Leeds United	3	1.1	51%	54%	142.8	149.0	1.04	16.70	20.00	1.20	4.15	4.56	1.10
Southampton	4	1.08	49%	54%	135.4	150.0	1.11	12.10	17.80	1.47	3.01	3.85	1.28
Bournemouth	3	1.07	44%	56%	137.4	144.1	1.05	14.30	15.80	1.11	4.00	3.40	0.85
Nottingham Forest	2	1.02	40%	56%	136.1	134.1	0.99	13.80	12.80	0.93	3.83	2.68	0.70
Burnley	4	0.96	43%	55%	140.0	147.2	1.05	12.80	16.00	1.25	2.78	3.04	1.09
Sheffield United	3	0.82	41%	58%	135.3	143.3	1.06	12.60	13.90	1.10	2.98	2.78	0.93
Luton Town	1	0.69	44%	55%	133.9	143.3	1.07	12.00	16.20	1.35	2.84	3.54	1.25
West Bromwich Albion	1	0.67	40%	56%	138.2	140.3	1.01	12.90	14.20	1.10	2.91	2.95	1.01
Watford	2	0.66	43%	55%	132.5	142.8	1.08	12.40	13.70	1.11	3.36	2.83	0.84
Norwich City	2	0.51	47%	57%	136.0	146.1	1.07	11.90	15.00	1.26	3.04	3.28	1.08

TD = team total distance (m), HID = high-intensity distance (m), SprD = sprint distance (m), TIP = team in-possession, TOOP = team out-of-possession

**TABLE 3:** The top six teams per season.

Position	2019	2020	2021	2022	2023
1	Liverpool	Manchester City	Manchester City	Manchester City	Manchester City
2	Manchester City	Manchester United	Liverpool	Arsenal	Arsenal
3	Manchester United	Liverpool	Chelsea	Manchester United	Liverpool
4	Chelsea	Chelsea	Tottenham Hotspur	Newcastle United	Aston Villa
5	Leicester City	Leicester City	Arsenal	Liverpool	Tottenham Hotspur
6	Wolverhampton Wanderers	West Ham United	Manchester United	Brighton and Hove Albion	Chelsea

Note: Wolverhampton Wanderers finished sixth in the 2019 season as games which finished 11 v 11 were only accounted for.

All statistical analysis was completed using R software (version 4.5.0, r-project.org), Rstatix (0.7.2) and tidyverse (2.0.0). Pearson product-moment correlation coefficient (*r*) was used to determine correlations between a team’s PPG over five seasons and running load (TD (m), HID (m), HSR (m) and SprD (m)) total, in and out-ofpossession, ball in play and possession percentage in a game. BiP time was taken directly from tracking data, representing the duration the ball was actively in play. Correlations were determined as trivial (< 0.10), small (0.10–0.29), moderate (0.30–0.49), large (0.50–0.69), very large (0.70–0.89), almost perfect (> 0.90–0.99) [27]. Statistical significance was set at p < 0.05.

To compare running load and possession variables between different levels of opposition, four comparisons were made to determine teams attributes: (1) T6 teams when playing other T6 teams, (2) T6 teams when playing teams ranked 7–20, (3) teams ranked 7–20 when playing T6, and (4) teams ranked 7–20 when playing other teams ranked 7–20. A Wilcoxon rank-sum test (Mann-Whitney U) was used to calculate effect size (ES) and *p*-value. The effect size is calculated as Z statistic divided by square root of the sample size and varies from 0 to close to 1. The interpretation for ES was 0.1–0.3 (small effect), 0.3–0.5 (moderate effect) and > = 0.5 (large effect) [28].

## RESULTS

### Running Load Possession Outputs in Comparison to Points Per Game

A very large positive correlation was found between PPG and possession percentage. Large positive correlations were found for TOOP/ TIP ratios across TD, HSR, HID and SprD as well as BiP time. Moderate positive correlations were observed for TOOP metrics TD, HSR, HID and SprD. SprD showed a small positive correlation. Contrastingly, TIP metrics were negatively correlated to PPG with smallto-moderate correlations for SprD, HID and HSR, with a large negative correlation for TD TIP (Table 4).

**TABLE 4:** Correlations (*r* ± 95% confidence intervals) between possession and running load variables to points per game over five seasons in the English Premier League (EPL).

Possession and Running Load Variables	*r: correlation to PPG*	lower	upper	p-value
Possession	0.74*	0.64	0.83	< 0.001
TD TOOP/ TIP Ratio	0.65*	0.52	0.75	< 0.001
HSR TOOP/ TIP Ratio	0.62*	0.47	0.72	< 0.001
HID TOOP/ TIP Ratio	0.59*	0.44	0.70	< 0.001
BiP	0.51*	0.34	0.64	< 0.001
SprD TOOP/ TIP Ratio	0.50*	0.34	0.63	< 0.001
HSR TOOP	0.45*	0.28	0.59	< 0.001
HID TOOP	0.44*	0.27	0.59	< 0.001
SprD TOOP	0.41*	0.23	0.56	< 0.001
TD TOOP	0.34*	0.14	0.49	< 0.001
SprD	0.25*	0.05	0.42	0.010
HID	0.17	-0.02	0.36	0.085
HSR	0.13	-0.07	0.31	0.149
TD	0.04	-0.16	0.24	0.570
SprD TIP	-0.22*	-0.40	-0.03	0.023
HID TIP	-0.37*	-0.53	-0.19	< 0.001
HSR TIP	-0.41*	-0.57	-0.24	< 0.001
TD TIP	-0.57*	-0.68	-0.41	< 0.001

* p < 0.05; TD = team total distance, HSR = high-speed running, HID = high-intensity distance, SprD = sprint distance, TIP = team in-possession, TOOP = team out-of-possession, BiP = Ball in Play

### Differences in Running Load and Technical Outputs between Top 6 Teams and Teams 7–20

Table 5 shows comparisons between running and technical metrics when playing a top 6 team versus teams 7–20.

**TABLE 5:** Comparison of differences in running load and technical outputs when playing teams who finished 1–6 (Top 6) versus teams who finished 7–20 (Teams 7–20) over five seasons in the English Premier League (EPL).

	Teams 1–6					Teams 7–20				

	vs Rest	vs Top Six	Difference Rest – Top 6	Effect size	P-value	vs Rest	vs Top Six	Difference Rest – Top 6	Effect size	P-value
Points per Game	2.2	1.4	-0.82	-0.66	< 0.001	1.4	0.6	-0.72	-0.61	< 0.001
Distance (m/min)	106	107	1.09	0.25	< 0.001	105	107	1.45	0.33	< 0.001
High-Intensity Distance (m/min)	8.3	8.7	0.41	0.39	< 0.001	8.2	8.3	0.15	0.13	0.005
High-Speed Running (m/min)	6.3	6.6	0.3	0.41	< 0.001	6.3	6.4	0.11	0.12	0.007
Sprint Distance (m/min)	1.9	2	0.11	0.27	< 0.001	1.8	1.9	0.04	0.11	0.02
Distance TIP (m/min)	130	132	2.13	0.3	< 0.001	135	136	1.01	0.16	< 0.001
High-Intensity Distance TIP (m/min)	11.5	12.3	0.77	0.27	< 0.001	12.8	13.2	0.46	0.17	< 0.001
High-Speed Running TIP (m/min)	8.5	9	0.45	0.25	< 0.001	9.5	9.8	0.25	0.13	0.003
Sprint Distance TIP (m/min)	3	3.3	0.31	0.27	< 0.001	3.2	3.4	0.21	0.19	< 0.001
Distance TOOP (m/min)	148	145	-2.35	-0.32	< 0.001	146	142	-4.12	-0.54	< 0.001
High-Intensity Distance TOOP (m/min)	17.6	16.8	-0.77	-0.2	0.006	16.3	14.8	-1.49	-0.39	< 0.001
High-Speed Running TOOP (m/min)	13.8	13.2	-0.62	-0.23	0.002	12.9	11.7	-1.19	-0.43	< 0.001
Sprint Distance TOOP (m/min)	3.8	3.7	-0.15	-0.11	0.11	3.4	3.1	-0.29	-0.24	< 0.001
Distance TOOP/TIP Ratio	1.1	1.1	-0.04	-0.4	< 0.001	1.1	1	-0.04	-0.55	< 0.001
High-Intensity Distance TOOP/TIP Ratio	1.7	1.5	-0.17	-0.28	< 0.001	1.3	1.2	-0.16	-0.4	< 0.001
High-Speed Running TOOP/TIP Ratio	1.7	1.6	-0.16	-0.29	< 0.001	1.4	1.2	-0.16	-0.42	< 0.001
Sprint Distance TOOP/TIP Ratio	1.5	1.3	-0.22	-0.22	0.001	1.2	1	-0.17	-0.28	< 0.001
Ball in Play (BiP) (% of time per game)	58%	59%	1%	0.19	0.007	55%	58%	3%	0.62	< 0.001
Possession (% per game)	59%	50%	-9%	-0.96	< 0.001	50%	41%	-9%	-1.04	< 0.001

TIP = team in-possession, TOOP = team out-of-possession

#### Top 6 teams

Playing other T6 teams was associated with lower PPG and possession (moderate effects). When facing T6 opponents, T6 teams recorded slightly lower TOOP running intensities (TD, HID, HSR; m · min^−1^) and lower TOOP/TIP ratios compared with their values against teams ranked 7–20. Small increases in BiP time, TIP running intensity and overall running load metrics when playing against T6 teams.

#### Teams 7–20

Lower PPG and reduced possession when playing T6 teams. TOOP (TD, HID, HSR) running metrics (m · min^−1^) and TOOP/TIP ratios were significantly lower (small-to-moderate effects). Moderate increases in BiP, and trivial increases in TIP running intensity and overall running load metrics, apart from distance (small increase) when playing against T6 teams.

## DISCUSSION

The aim of this study was to analyse the relationships between possession and running load metrics in relation to team success, and to determine whether a more pronounced difference exists between top and lower-ranked teams in the EPL. The main findings were; (1) possession is strongly associated with success, (2) greater out-of-possession and lower in-possession running per minute are related to higher PPG, (3) both T6 and lower-ranked teams show lower in-possession intensity and higher total running load outputs when playing T6 teams, (4) SprD is the only total running load metric that was directly associated with PPG. These results expand on previous research by applying per-minute in-possession and out-of-possession analyses across five EPL seasons.

### Ball possession is key to success

Possession had a very large correlation to PPG (*r* = 0.74; *p < 0*.001), supporting previous literature in the EPL [29] and other leagues [9, 30] where more successful teams had higher ball possession. Greater possession and BiP time, may allow teams to have more attempts on goal [7], which in turn increases chances of match success [9]. T6 teams averaged 59% possession over the five seasons, consistent with Poli et al. [8] One possible explanation for these findings is that stronger teams generally possess more technically proficient players [31], allowing them to maintain possession more effectively and, in turn, create more opportunities to progress up the pitch and generate chances.

Recent studies show that leading teams (scoreline) tend to have greater ball possession [6, 32], contrasting earlier research which reported greater possession for losing teams [33–34]. This may indicate a shift in strategic preferences, with teams increasingly adopting a possession-based style of play [35]. However, ball possession alone is unlikely to determine the outcome of a match. Its impact is closely tied to the location of possession, being more advantageous the closer it is to the opponent’s goal [6, 9], and the effectiveness of player movements to create space. This suggests that the value of possession lies not just in quantity, but in where and how it is utilized, highlighting an important area for further exploration.

### Distances out-of-possession are highly significant

Analysing running outputs per minute in-possession and out-ofpossession, rather than relying solely on total distances, a significant association emerges between TOOP running and PPG (Table 4). TD-TOOP (r = .34, p < 0.001), HID-TOOP (r = .44, p < 0.001), HSR-TOOP (r = .45, p < 0.001), and SprD-TOOP (r = .41, p < 0.001) all showed positive relationships with PPG, which strengthened when running variables were expressed as TOOP/TIP ratios (Table 4). These findings indicate that successful teams tend to exert greater relative intensity out-of-possession than in-possession.

This may reflect the behaviour of stronger teams, who aim to dominate possession, regaining it quickly when they lose it. More technically skilled players [31], enable teams to employ more aggressive TOOP strategies like counterpressing, which is the pressure placed upon the opposition after losing the ball [36]. Quick regains closer to the oppositions goal increase chances of scoring [37], and are also linked to defensive success [38], whilst allowing opposition teams to have the ball for longer lead to increased chances of conceding [39]. Teams typically maintain possession for longer durations when facing weaker opposition (an increase in 9 seconds mean ball possession duration) [23].

Relative intensity findings suggest higher-possession teams may enter defensive transitions in a less fatigued state. Modric et al. [40] showed that teams with less possession are often required to run more, suggesting that possession dominant teams may be better at producing higher intensity efforts immediately after ball loss (TOOP metrics per min), potentially increasing successful regains. Regain success is further amplified if ball loss occurs closer to the opposition goal [41], a characteristic of more successful teams [42]. Tactical trends shifted from counter-attacking styles [43] towards possession-based play and high pressing [35, 44]; rapid defensive organisation and compactness around the ball have become increasingly important for ball regain success [24, 45].

Although correlations cannot confirm causation, the TOOP patterns likely reflect the underlying qualities of stronger teams. High running outputs alone do not explain performance; rather, technically and tactically skilled players who coordinate intelligent movements within cohesive tactical structures give these efforts value [3, 22]. TOOP intensity is better viewed as a by-product of high-quality players operating within well-organised tactical structures, not an isolated driver of success. Thus it represents the expression of tactical and technical superiority rather than isolated physical effort.

### Running in-possession is negatively correlated to points per game

Successful teams demonstrated negative correlations between TIP running metrics and PPG (Table 4). This likely suggests successful teams favour a slower build up style, once possession is regained, potentially linked to opposition set up, especially against deeper defensive blocks. TOOP metrics drop when playing against a T6 team (Table 5), which suggests that opposition teams are more likely to sacrifice possession and adopt deeper defensive blocks to protect the space around their own goal. In these scenarios, the attacking team is often required to be more patient in-possession, leading to a reduction in HSR and SprD distances attained due to closer proximity to the opposition goal limits the space for extended high-intensity efforts.

Research has shown that teams respond dynamically to the behaviour of their opponents, adjusting their tactical choices in real time [4, 40, 46]. When facing compact defensive formations, top teams may prioritise structured possession, positional play, and short passing networks over relying on high-speed transitions. Although faster actions such as overlapping runs may still occur, they are likely to take place over shorter distances when the opposition adopts a deeper, more compact setup (low block) [21]. Thus, high physical output while in-possession may not necessarily indicate success, especially in matches where the opposing team’s main objective is to reduce space and slow the pace of the game. In these situations, maintaining possession and wearing down the opponent through technical superiority becomes a more effective strategy than relying on repeated high-speed runs. As such, the physical profile in-possession may be characterised more by sharp accelerations over short distances, such as positional rotations, and movement to create angles rather than large volume of high-speed output, possibly linked to smaller spaces [47]. Furthermore, match context is crucial to consider. Teams that are leading tend to control the tempo and adopt a lower-risk approach, particularly when in-possession, which may explain the negative correlation between TIP metrics and PPG. A possession-based strategy, especially when ahead, often focuses on maintaining stability and minimising turnovers, thereby reducing the necessity for frequent high-intensity movements with the ball.

### Differences in possession and running load when facing top-six teams

Significant differences in possession and running behaviours were observed when teams faced T6 opposition. Both T6 and teams ranked 7–20 earned fewer points against T6 opponents, highlighting the strength of T6 teams. Teams also ran more (m/min) against T6 opponents, including the T6 teams themselves, consistent with evidence that EPL teams tend to match opponents’ running outputs [4].

Interestingly, teams ran more per minute in-possession when facing T6 opponents, likely reflecting reduced overall possession and increased reliance on transitional counterattacks, consistent with findings that low-possession teams run more in-possession [48–49]. Conversely, TOOP running per minute decreased, particularly for teams 7–20, suggesting the use of deeper, more passive defensive blocks against superior opponents [50]. Such blocks require fewer high-speed actions and more lateral shifting [40], prioritising protection of central areas [45].

BiP time increased during matches against T6 teams, likely due to longer attacking phases and fewer interruptions. Possession for both groups decreased by 9% when facing T6 teams, consistent with the T6’s greater technical capacity to retain the ball [31].

These findings reinforce the need to interpret running data in context, with opposition strength being a critical variable. Running load output is not fixed, it adapts based on the tactical needs of each match. For practitioners, this underscores the importance of planning training and recovery strategies with opponent profile in mind and ensuring match preparation reflects the likely demands imposed by opposition quality.

### Total running load (TD, HID, HSR, SprD) has a reduced significance to success

Total running load (TD, HID, HSR and SprD) showed a reduced association to success, contrasting with earlier findings from the same authors, that HID, HSR and SprD were associated to success [4]. This reduced significance reflects the continually evolving nature of the game, where physical trends and tactical approaches shift season-to-season. While previous research identified small yet significant correlations between running loads covered and final league position [4], the current data shows this link has reduced. From the present analysis, only SprD maintained a statistically significant relationship with points gained (Table 4; *r* = .25; *p* = 0.01), suggesting that it is not the overall volume of running that differentiates teams, it is the ability to execute explosive actions at critical moments.

This may reflect a levelling in physical output across the league. Teams may now be matching each other more closely in total running output, understanding the importance of meeting the physical demands of modern football. Closing this physical gap could be a deliberate tactic by lower-ranked teams to reduce the time and space available to higher-quality opposition, a principle tied to effective space management and compact defensive structures. In turn, this might reduce opportunities for elite teams to exploit technical superiority, especially in open play. Such a shift could suggest a rise in man-oriented or hybrid pressing systems, where individual players are tasked with tightly tracking their opponents throughout the match. By maintaining proximity and preventing unmarked movement, teams reduce their vulnerability to overloads and counterattacks. Recent research by Plakias et al. [51] and Forcher et al. [45], reinforces this, showing that teams who effectively prevent counterattacks and remain compact around the ball are more likely to succeed.

Counterattacks are known to generate some of the highest intensity running actions in football, particularly in terms of high-speed running and sprints [52–53]. Despite this, the declining correlation between total running volume and points suggests that performance staff and coaches must increasingly focus on *when and where* running is most effective. This places greater emphasis on the tactical application of physical effort rather than sheer volume. Overall, the evolution noted here identifies the importance of integrating physical performance data with tactical context. Head coaches, analysts, and sports scientists must work together to identify the specific phases and locations in which running contributes most directly to performance, such as regaining possession high up the pitch, exploiting a disorganised back line, or creating overloads in transition. It is within these decisive moments that sprinting and high-speed actions may carry more influence than distance covered over a game. To understand this context further, it is important to gain the knowledge of head coaches and their coaching team to understand what the key moments of a game are from a physical perspective to fully determine efficient running for a player.

### Limitations and future research directions

The current study only investigated ‘extensive’ running load outputs, due to nature of those reported by current generation optical tracking systems. Currently, mechanical orientated outputs which characterise the more ‘intensive’ running load demands (accelerations and decelerations) have yet been validated. Accelerations and decelerations are essential for executing rapid offensive and defensive manoeuvres in football and occur frequently during matches [54]. Therefore, future research should explore the impact of these movements on team success in the EPL and examine how they vary between top-ranked and lower-ranked teams. It must be noted, the analysis used team-season averages and correlation-based methods, the results reflect between-team associations rather than within-team longitudinal effects. Mixed-effects modelling may yield different estimates if the research question concerned within-team changes over time.

A further limitation is the exclusion of several contextual variables known to influence physical and tactical behaviour, including game state, formation, opponent strength, match location, and ball-in-play time [3, 21–22]. Although opponent strength was explored descriptively through T6 comparisons, these factors were not incorporated analytically and may partly explain variation in running intensities. Future research should integrate contextual and tactical covariates to isolate their contribution to possession-related running demands.

Additionally, the trends in this paper are over a five-season period. Findings of the current study suggest trends are evolving over time, with total running loads now becoming less significant in comparison to recent studies [4]. Given the constantly evolving nature of football, it is recommended that teams regularly update their understanding of trends to anticipate the short-term direction of the game and better prepare their squads. No study to date has examined the evolution of accelerations and decelerations in the EPL, despite suggestions that these actions may become increasingly important as tactical aspects of the game continue to develop [54, 55].

### Practical Implications

#### Use TOOP/TIP Ratios to Inform Playing Style and Training Load

The TOOP/TIP ratio could be used for understanding the balance between a team’s intensity with and without the ball. Teams that rely on structured build up in-possession and pressing, quick ball recovery out-of-possession should see higher TOOP/TIP values. This may help design conditioning strategies that aim to focus on football specific actions that support a team’s playing philosophy. Aligning physical conditioning with tactical goals is essential. Rather than isolating running, training drills should therefore simulate match scenarios where physical actions carry direct tactical consequences, e.g., recovering after losing possession, or pressing to force mistakes. This reinforces the purpose of the run and likely enhances decisionmaking under fatigue.

#### Train intensity out-of-possession

From the findings in the current study TOOP is strongly linked to success. To assist with conditioning, creating drills that see transitional moments (i.e. reaction to ball loss) occur more frequently, may be able to help from a tactical as well as a physical aspect. Ensuring players react to ball loss and increase pressure on the opponent may improve a team’s opportunity to capitalise on opponent disorganisation.

#### Evaluate Sprinting Output in Key Moments, Not Just Volume

Since sprint distance totals are one of the few running load metrics still linked to success and are supported by the findings in the current study. Coaches should therefore ensure that players can produce sprinting actions at key moments in a match, particularly when these actions contribute directly to decisive tactical outcomes. It is suggested for coaches and performance staff to determine *when and where* they want the players to sprint in their game model and apply it within drill design, including conditioning. In practice, these moments often include pressing triggers, recovery runs following ball loss, counterattacking movements, overlaps and underlaps in wide areas, runs in behind the defensive line, and defensive transition actions where rapid acceleration is essential to delay or disrupt the opponent.

#### Contextualise Running Load in Pre and Post-Match Feedback

Practitioners and coaches, based on the findings of the current study, should avoid comparing running metrics across matches without accounting for game state, opposition strategy, and tactical role. Table 5 highlights the effects that opposition quality can have on a team. Providing context is likely to help plan football specific conditioning drills that can benefit the team in preparing for a game. Moreover, understanding how running demands systematically shift when facing T6 opponents can support more accurate load interpretation and inform match-specific preparation, ensuring that feedback reflects tactical context rather than running alone.

## CONCLUSIONS

The findings in this study suggest that running performance in football is deeply context-dependent and not a straightforward predictor of success. While running load TOOP show moderate correlations with PPG, total running load alone offers limited explanatory power. Top performing teams consistently demonstrate lower physical output in-possession, suggesting a favouring for control, structure, and positional dominance, while maintaining high intensity out-of-possession to support pressing and rapid ball recovery, thus increasing their chances of having more ball possession. Moreover, both T6 and lower-ranked teams adjust their physical profiles significantly based on opponent quality, with running load, possession share, and ballin-play time all shifting depending on the tactical demands imposed by elite opposition. These patterns reflect the modern game’s complexity, where physical performance must be evaluated not in isolation, but through the lens of tactical intent, match context, and opponent strategy. Coaches, analysts, and practitioners must continue to evolve how they interpret and utilise running data, to measure effort and simultaneously understand how and when physical actions contribute most effectively to team success.

## Key Findings

► Possession is highly correlated to success
► Points per game are correlated with high out-of-possession and low in-possession running load
► Teams run more overall totals and in-possession and less out-of-possession against top 6 teams
► Larger differences are seen for teams 7–20 than top 6 teams when playing teams 7–20 vs top 6 teams
► Total running load has reduced its correlation to success. Only sprint distance is correlated to points per game

## Additional

This paper was published with the permission of The Premier League but not implying the views of The Premier League.

## References

[1] Andrzejewski M, Oliva-Lozano JM, Chmura P, et al. Analysis of team success based on match technical and running performance in a professional soccer league. BMC Sports Sci Med Rehabil. 2022;14(1):82. doi:10.1186/s13102-022-00473-y 35513858 PMC9069775

[2] Ju W, Hawkins R, Doran D, et al. Tier-specific contextualised high-intensity running profiles in the English Premier League: more on-ball movement at the top. Biol Sport. 2023;40(2):561-573. 37077798 10.5114/biolsport.2023.118020 PMC10108770

[3] Morgans R, Mandorino M, Beato M, et al. Contextualized high-speed running and sprinting during English Premier League match-play with reference to possession, positional demands and opponent ranking. Biol Sport. 2025;42(3):119-127. 40657003 10.5114/biolsport.2025.147011 PMC12244393

[4] Allen T, Taberner M, Zhilkin M, Alexander J, Harper D, Rhodes D. Is it as simple as run more, win more? Influence of running load on match outcome and final league position across nine consecutive English Premier League football seasons. Int J Sports Sci Coach. Published online August 29, 2025. doi:10.1177/17479541251371004

[5] Casal CA, Anguera MT, Maneiro R, Losada JL. Possession in football: more than a quantitative aspect—a mixed method study. Front Psychol. 2019;10:1176. doi:10.3389/fpsyg.2019.01176 30936844 PMC6431675

[6] Casal CA, Maneiro R, Ardá T, Marí FJ, Losada JL. Possession zone as a performance indicator in football: the game of the best teams. Front Psychol. 2017;8:1176. doi:10.3389/fpsyg.2017.01176 28769833 PMC5509942

[7] Hughes M, Franks I. Analysis of passing sequences, shots and goals in soccer. J Sports Sci. 2005;23(5):509-514. 16194998 10.1080/02640410410001716779

[8] Poli DR, Ravenel L, Besson R. CIES Football Observatory Monthly Report n°41 – January 2019. CIES; 2019.

[9] Collet C. The possession game? A comparative analysis of ball retention and team success in European and international football, 2007–2010. J Sports Sci. 2013;31(2):123-136. 23067001 10.1080/02640414.2012.727455

[10] Zambom-Ferraresi F, Rios V, Lera-López F. Determinants of sport performance in European football: what can we learn from the data? Decis Support Syst. 2018;114:18-28.

[11] Brîndescu S, Datcu FR, Buda IA. Study on the efficiency of advanced pressing in the Premier League. Balt J Health Phys Act 2021;13(spec):115-122.

[12] Allen T, Taberner M, Zhilkin M, Rhodes D. Running more than before? The evolution of running load demands in the English Premier League. Int J Sports Sci Coach. 2024;19(2):779-787.

[13] Allen T, Taberner M, Zhilkin M, Green M. Evolving running load demands and fixture congestion in the English Premier League: a decade of insights from 2015/16 to 2024/25. Br J Sports Med. In review. 10.1136/bjsports-2025-110430 PMC12772561 41419309

[14] Chmura P, Konefał M, Chmura J, et al. Match outcome and running performance in different intensity ranges among elite soccer players. Biol Sport. 2018;35(2):197-203. 30455549 10.5114/biolsport.2018.74196 PMC6234309

[15] Teixeira JE, Forte P, Ferraz R, et al. Integrating physical and tactical factors in football using positional data: a systematic review. PeerJ. 2022;10:e14381. doi:10.7717/peerj.14381 36405022 PMC9671036

[16] Bradley PS, Sheldon W, Wooster B, et al. High-intensity running in English FA Premier League soccer matches. J Sports Sci. 2009;27(2):159-168. 19153866 10.1080/02640410802512775

[17] Brito Souza D, López-Del Campo R, Blanco-Pita H, Resta R, Del Coso J. Association of match running performance with and without ball possession to football performance. Int J Perform Anal Sport. 2020;20(3):483-494.

[18] Rampinini E, Impellizzeri FM, Castagna C, Coutts AJ, Wisløff U. Technical performance during soccer matches of the Italian Serie A league: effect of fatigue and competitive level. J Sci Med Sport. 2009;12(1):227-233. 18083631 10.1016/j.jsams.2007.10.002

[19] Hoppe MW, Slomka M, Baumgart C, Weber H, Freiwald J. Match running performance and success across a season in German Bundesliga soccer teams. Int J Sports Med. 2015;36(7):563-566. 25760152 10.1055/s-0034-1398578

[20] Modric T, Versic S, Drid P, et al. Analysis of running performance in the offensive and defensive phases of the game: is it associated with the team achievement in the UEFA Champions League? Appl Sci. 2021;11(18):8765. doi:10.3390/app11188765

[21] Jerome BWC, Stoeckl M, Mackriell B, Dawson CW, Fong DTP, Folland JP. Evidence for a new model of the complex interrelationship of ball possession, physical intensity and performance in elite soccer. Scand J Med Sci Sports. 2024;34(1):e14546. 38059701 10.1111/sms.14546

[22] Morgans R, Radnor J, Fonseca J, et al. Match running performance is influenced by possession and team formation in an English Premier League team. Biol Sport. 2024;41(3):275-286. 10.5114/biolsport.2024.135414 PMC11167476 38952911

[23] Gonçalves B, Coutinho D, Exel J, et al. Extracting spatial-temporal features that describe a team match demands when considering the effects of the quality of opposition in elite football. PLoS One. 2019;14(8):e0221368. doi:10.1371/journal.pone.0221368 31437220 PMC6705862

[24] Forcher L, Forcher L, Altmann S, Jekauc D, Kempe M. The success factors of rest defense in soccer – a mixed-methods approach of expert interviews, tracking data, and machine learning. J Sports Sci Med. 2023;22(4):707-725. 38045740 10.52082/jssm.2023.707 PMC10690503

[25] FIFA. FIFA Quality Performance Reports for EPTS. Published 2019. Accessed August 15, 2022. https://www.fifa.com/technical/football-technology/standards/epts/fifa-quality-performance-reports-for-epts

[26] Mikikis D, Michailidis Y, Mandroukas A, Mavrommatis G, Metaxas T. Goalkeeper performance: analysis of goalkeepers’ contribution to their team’s build-up under the opponent’s pressure in the 2018 World Cup. Cent Eur J Sport Sci Med. 2021;34(2):77-86.

[27] Hopkins WG, Marshall SW, Batterham AM, Hanin J. Progressive statistics for studies in sports medicine and exercise science. Med Sci Sports Exerc. 2009;41(1):3-13. doi:10.1249/MSS.0b013e31818cb278 19092709

[28] Tomczak M, Tomczak E. The need to report effect size estimates revisited: an overview of some recommended measures of effect size. Trends Sport Sci. 2014;21(1):19-25.

[29] Parziale E, Yates P. Keep the ball! The value of ball possession in soccer. Reinvention. 2013;6(1).

[30] Kempe M, Vogelbein M, Memmert D, Nopp S. Possession vs. direct play: evaluating tactical behavior in elite soccer. Int J Sports Sci. 2014;4(6A):35-41.

[31] Liu T, Yang L, Chen H, García-de-Alcaraz A. Impact of possession and player position on physical and technical-tactical performance indicators in the Chinese Football Super League. Front Psychol. 2021;12:722200. doi:10.3389/fpsyg.2021.722200 34659035 PMC8511401

[32] Maneiro R, Casal CA, Álvarez I, et al. Offensive transitions in high-performance football: differences between UEFA Euro 2008 and UEFA Euro 2016. Front Psychol. 2019;10:1230. doi:10.3389/fpsyg.2019.01230 31275190 PMC6591362

[33] Jones PD, James N, Mellalieu SD. Possession as a performance indicator in soccer. Int J Perform Anal Sport. 2004;4(1):98-102.

[34] Lago C, Martín R. Determinants of possession of the ball in soccer. J Sports Sci. 2007;25(9):969-974. 17497397 10.1080/02640410600944626

[35] Barreira D, Garganta J, Castellano J, Machado J, Anguera MT. How elite-level soccer dynamics has evolved over the last three decades? Input from generalizability theory. Cuad Psicol Deporte. 2015;15(1):51-62.

[36] Fernández Navarro FJ. Analysis of Styles of Play in Soccer and Their Effectiveness. [PhD thesis]. University of Portsmouth; 2018. Accessed March 1, 2023. https://api.semanticscholar.org/CorpusID:150392656

[37] Tenga A, Holme I, Ronglan LT, Bahr R. Effect of playing tactics on goal scoring in Norwegian professional soccer. J Sports Sci. 2010;28(3):237-244. 20391095 10.1080/02640410903502774

[38] Vogelbein M, Nopp S, Hökelmann A. Defensive transition in soccer – are prompt possession regains a measure of success? A quantitative analysis of German Fußball-Bundesliga 2010/2011. J Sports Sci. 2014;32(11):1076-1083. 24506111 10.1080/02640414.2013.879671

[39] Shafizadeh M, Lago-Penas C, Gridley A, Platt GK. Temporal analysis of losing possession of the ball leading to conceding a goal: a study of the incidence of perturbation in soccer. Int J Sports Sci Coach. 2014;9(4):627-636.

[40] Modric T, Versic S, Morgans R, Sekulic D. Match running performance characterizing the most elite soccer match-play. Biol Sport. 2023;40(4):949-958. 37867756 10.5114/biolsport.2023.124847 PMC10588580

[41] Fernandez-Navarro J, Ruiz-Ruiz C, Zubillaga A, Fradua L. Tactical variables related to gaining the ball in advanced zones of the soccer pitch: analysis of differences among elite teams and the effect of contextual variables. Front Psychol. 2019;10:3040. doi:10.3389/fpsyg.2019.03040 32038403 PMC6985566

[42] González-Rodenas J, Ferrandis J, Moreno-Pérez V, et al. Differences in playing style and technical performance according to the team ranking in the Spanish football LaLiga: a thirteen seasons study. PLoS One. 2023;18(10):e0293095. 37862370 10.1371/journal.pone.0293095 PMC10588845

[43] Bate R. Football chance: tactics and strategy. In: Science and Football. Routledge; 1988.

[44] Forcher L, Forcher L, Altmann S, Jekauc D, Kempe M. The keys of pressing to gain the ball – characteristics of defensive pressure in elite soccer using tracking data. Sci Med Footb. 2024;8(2):161-169. 36495564 10.1080/24733938.2022.2158213

[45] Forcher L, Forcher L, Altmann S, Jekauc D, Kempe M. Is a compact organization important for defensive success in elite soccer? – Analysis based on player tracking data. Int J Sports Sci Coach. 2024;19(2):757-768.

[46] Frencken W, Lemmink K, Delleman N, Visscher C. Oscillations of centroid position and surface area of soccer teams in small-sided games. Eur J Sport Sci. 2011;11(4):215-223.

[47] Silva H, Nakamura FY, Beato M, Marcelino R. Acceleration and deceleration demands during training sessions in football: a systematic review. Sci Med Footb. 2023;7(3):198-213. 35700979 10.1080/24733938.2022.2090600

[48] Pan P, Lago-Peñas C, Lorenzo-Martinez M, et al. Interaction effects between possession status and percentage: insights from modeling match-running performance across possession status in male soccer. Biol Sport. 2025;43(1):35-44. 41668939 10.5114/biolsport.2025.151653 PMC12884881

[49] Asian-Clemente J, Suarez-Arrones L, Requena B, Santalla A. Influence of tactical behaviour on running performance in the three most successful soccer teams during the competitive season of the Spanish first division. J Hum Kinet. 2022;82:135-144. 36196354 10.2478/hukin-2022-0040 PMC9465736

[50] Gollan S, Bellenger C, Norton K. Contextual factors impact styles of play in the English Premier League. J Sports Sci Med. 2020;19(1):78-83. 32132830 PMC7039037

[51] Plakias S, Tsatalas T, Armatas V, Tsaopoulos D, Giakas G. Tactical situations and playing styles as key performance indicators in soccer. J Funct Morphol Kinesiol. 2024;9(2):88. 38804454 10.3390/jfmk9020088 PMC11130910

[52] Bortnik L, Burger J, Rhodes D. The mean and peak physical demands during transitional play and high pressure activities in elite football. Biol Sport. 2022;39(4):1055-1064. 36247966 10.5114/biolsport.2023.112968 PMC9536371

[53] Faude O, Koch T, Meyer T. Straight sprinting is the most frequent action in goal situations in professional football. J Sports Sci. 2012;30(7):625-631. 22394328 10.1080/02640414.2012.665940

[54] Harper DJ, Carling C, Kiely J. High-intensity acceleration and deceleration demands in elite team sports competitive match play: a systematic review and meta-analysis of observational studies. Sports Med. 2019;49(12):1923-1947. 31506901 10.1007/s40279-019-01170-1 PMC6851047

[55] Harper DJ, Sandford GN, Clubb J, et al. Elite football of 2030 will not be the same as that of 2020: what has evolved and what needs to evolve? Scand J Med Sci Sports. 2021;31(2):493-494. 33483977 10.1111/sms.13876

